# Acceptance of illness and its relationship with quality of life in women after mastectomy

**DOI:** 10.1192/j.eurpsy.2026.10763

**Published:** 2026-07-31

**Authors:** D. Schneider-Matyka, K. Rachubińska, J. Kopeć, M. Stanisławska, K. Augustyniuk, E. Grochans, A. Cybulska

**Affiliations:** 1 Department of Nursing; 2 Scientific Club at the Department of Nursin, Pomeranian Medical University in Szczecin, Szczecin, Poland

## Abstract

**Introduction:**

Breast cancer is the most common malignancy among women worldwide. Mastectomy, while often life-saving, significantly affects physical, emotional, and social functioning. Acceptance of illness is recognized as a key psychological resource that may facilitate adaptation, reduce symptom burden, and improve health-related quality of life (HRQoL).

**Objectives:**

To assess the relationship between the level of disease acceptance and quality of life in women after mastectomy, with consideration of sociodemographic determinants of acceptance.

**Methods:**

A cross-sectional, descriptive study was conducted in 2024 among 125 women after mastectomy at the West Pomeranian Oncology Center and the Amazon Association “AGATA” in Szczecin, Poland. Data were collected using a sociodemographic questionnaire, the Acceptance of Illness Scale (AIS), and the EORTC QLQ-C30 and QLQ-BR23 questionnaires. Statistical analyses included descriptive statistics, Mann–Whitney U, Kruskal–Wallis tests, and Spearman’s correlations, with significance set at p<0.05.

**Results:**

The mean AIS score was 28.8 (SD=7.05). Higher acceptance was significantly associated with better global quality of life and improved physical, emotional, cognitive, and social functioning (all p<0.001). It was negatively correlated with fatigue, pain, insomnia, appetite loss, constipation, and financial difficulties. In the breast cancer-specific module, greater acceptance was linked to a more positive body image, higher sexual enjoyment, and a better future perspective, and negatively correlated with systemic therapy side effects, breast and arm symptoms, and distress related to hair loss. Sociodemographic determinants of higher acceptance included higher education (p=0.002), better financial status (p=0.019), and being professionally active or retired compared with pensioners (p=0.024). Age, marital status, and living arrangement showed no significant associations.

**Conclusions:**

Acceptance of illness significantly improves the quality of life in women after mastectomy, enhancing multiple domains of functioning while reducing the severity of treatment-related symptoms. Its level is influenced by sociodemographic factors such as education, financial situation, and employment status.These findings highlight important practical implications: interventions aimed at strengthening disease acceptance and addressing social determinants should be integrated into psycho-oncological care and rehabilitation to support adaptation, reduce symptom burden, and improve long-term well-being in breast cancer survivors.

**Disclosure of Interest:**

None Declared

